# Itch in Psoriasis

**Published:** 1985-04

**Authors:** Clive E. H. Grattan

**Affiliations:** Registrar in Dermatology, Bristol Royal Infirmary, Bristol

## Abstract

Pruritus was assessed in plaque psoriasis, atopic eczema and acne vulgaris using visual analogue scales. The results demonstrate that itch is a common and frequently troublesome symptom in plaque psoriasis. This observation should be more widely recognised in textbooks and publications.


### Itch in Psoriasis

**Clive E. H. Grattan, M.B., B.Chir., M.R.C.P.**
Registrar in Dermatology, Bristol Royal Infirmary, Bristol

### ABSTRACT

Pruritus was assessed in plaque psoriasis, atopic eczema and acne vulgaris using visual analogue scales. The results demonstrate that itch is a common and frequently troublesome symptom in plaque psoriasis. This observation should be more widely recognised in textbooks and publications.

### INTRODUCTION

Psoriasis is derived from the Greek word psora, meaning itch or contagious skin condition. However, no mention is made of pruritus as a symptom in many dermatology textbooks and some publications uphold the widely taught view that it is a non-itchy dermatosis.[1,2] Clinical experience suggests that variable pruritus is common and may be troublesome. This study was undertaken to compare the severity of itch in plaque psoriasis, with atopic eczema and acne vulgaris as positive and negative controls.

### PATIENTS AND METHODS

Sixty consecutive inpatients (32 males, 28 females, mean age 39.4 years), admitted for treatment of stable chronic plaque psoriasis affecting more than 5% of the body surface area, completed a 10 cm visual analogue scale indicating severity of itch at their best and at their worst.

Forty-seven inpatients with active atopic eczema (25 male, 22 female, mean age 23.5 years) and 28 female outpatients, who were participating in a trial of treatment for acne vulgaris, also completed the visual analogue scales and acted as control groups.

The visual analogue scales were analysed by measuring from the base in centimetres and grouping the scores from one to ten. Scores for the three groups were compared by the two-tailed Mann–Whitney U non-parametric test.

### RESULTS

The mean itch scores for patients with psoriasis were greater than those with acne vulgaris but less than those with atopic eczema. The scoring distributions between psoriasis and the control groups were significantly different in each category (Table 1).

Taking an itch score of greater than 5 as a measure of troublesome pruritus, 75% of the psoriatics, 98% of the atopics and 7% of the acne group recorded troublesome pruritus when at their worst.

As the controls were not sex-matched, comparisons were made between the scoring distributions of the two sexes in psoriasis and atopic eczema. No significant difference was revealed (Mann–Whitney U test).

### DISCUSSION

These results indicate that pruritus is a common symptom in patients with moderate to extensive

**Table 1**

**Itch scores from visual analogue scales**

| | No. of patients | Itch scores: Category | Mean | Range | Probability* |
|---|---|---|---|---|---|
| Psoriasis | 60 | Best | 2.48 | (1–9) | |
| | | Worst | 7.15 | (1–10) | |
| Atopic eczema | 47 | Best | 3.36 | (1–10) | <0.05 |
| | | Worst | 9.32 | (3–10) | <0.001 |
| Acne vulgaris | 28 | Best | 1.21 | (1–5) | <0.001 |
| | | Worst | 2.25 | (1–9) | <0.001 |

*Mann–Whitney U two-tailed test with ties correction. Psoriasis vs. control.

*continued on page 42*

**Figure 5**

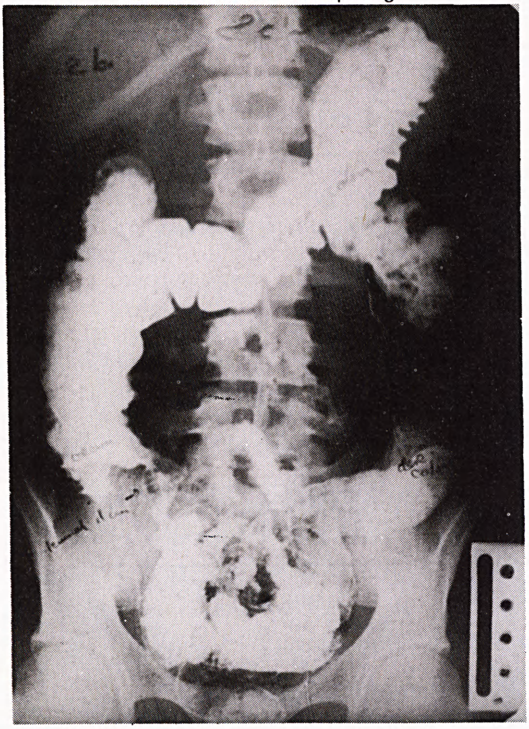

Conical caecum with spiking generic tissue response which may indeed be evoked by a wide variety of agents.

### REFERENCES

1. Crohn, B. B., Ginzburg, L. and Oppenheimer, G. B. (1932) *J.A.M.A.*, **99**, 1323.
2. Anand, S. S. (1956) *Anns. R.C.S. Eng.*, **19**, 205–222.

*continued from page 36*

plaque psoriasis and may be severe. This observation, which is well known to clinicians, should be more widely recognised in the literature.

### ACKNOWLEDGEMENTS

I thank Dr. F. Wojnarowska for organising the acne patients.

### REFERENCES

1. MARKS, R., Psoriasis. Martin Dunitz, Positive Health Guide, p. 17, 1981.
2. LOMHOLT, G., Psoriasis. Copenhagen, GEC gad, p. 68, 1963.

